# Observer agreement paradoxes in 2x2 tables: comparison of agreement measures

**DOI:** 10.1186/1471-2288-14-100

**Published:** 2014-08-28

**Authors:** Viswanathan Shankar, Shrikant I Bangdiwala

**Affiliations:** 1 Division of Biostatistics, Department of Epidemiology and Population Health, Albert Einstein College of Medicine, Bronx, NY 10461, USA; 2 Department of Biostatistics, Gillings School of Global Public Health, University of North Carolina, Chapel Hill, NC 27599, USA

**Keywords:** Rater agreement, 2x2 table, Cohen’s kappa, Aickin’s alpha, B-statistic, Delta, AC1-index

## Abstract

**Background:**

Various measures of observer agreement have been proposed for 2x2 tables. We examine the behavior of alternative measures of observer agreement for 2x2 tables.

**Methods:**

The alternative measures of observer agreement and the corresponding agreement chart were calculated under various scenarios of marginal distributions (symmetrical or not, balanced or not) and of degree of diagonal agreement, and their behaviors are compared. Specifically, two specific paradoxes previously identified for kappa were examined: (1) low kappa values despite high observed agreement under highly symmetrically imbalanced marginals, and (2) higher kappa values for asymmetrical imbalanced marginal distributions.

**Results:**

Kappa and alpha behave similarly and are affected by the marginal distributions more so than the B-statistic, AC1-index and delta measures. Delta and kappa provide values that are similar when the marginal totals are asymmetrically imbalanced or symmetrical but not excessively imbalanced. The AC1-index and B-statistics provide closer results when the marginal distributions are symmetrically imbalanced and the observed agreement is greater than 50%. Also, the B-statistic and the AC1-index provide values closer to the observed agreement when the subjects are classified mostly in one of the diagonal cells. Finally, the B-statistic is seen to be consistent and more stable than kappa under both types of paradoxes studied.

**Conclusions:**

The B-statistic behaved better under all scenarios studied as well as with varying prevalences, sensitivities and specificities than the other measures, we recommend using B-statistic along with its corresponding agreement chart as an alternative to kappa when assessing agreement in 2x2 tables.

## Background

Several measures of inter- and intra-rater agreement have been proposed over the years. Excellent reviews of such methods for both categorical and continuous variables are given in Banerjee *et al.*[1], Kramer *et al.*[2] and Landis *et al.*[3]. Cohen’s kappa [4] is the most commonly used index to assess concordance or agreement between two raters classifying units into discrete categories. Concordance is a term used to mean agreement in classification between the raters. When a single rater is being compared against a gold standard, agreement is also called ‘validity’ , while if a rater is being compared to another rater as in the absence of a gold standard, agreement is often also called ‘reliability’. Kappa corrects for chance agreement and is estimated by

$$\hat{k}=\frac{P_{o}-P_{e}}{1-P_{e}}\;\text{,}$$

where P_o_ is the proportion of overall observed agreement and P_e_ is the proportion of overall chance-expected agreement. The kappa statistic thus ranges between – P_e_ / (1-P_e_) to 1.

Kappa’s behavior has been questioned and its use debated for 2 × 2 tables [5-11]. The major concern is that its behavior is subject to changes in prevalence [9,11]. In addition, there are two paradoxes discussed by Feinstein and Cicchetti [7] related to the effect on kappa of the balance and symmetry of the marginal distributions. In the generic 2x2 table (Table 1), balance refers to whether the ratio of column marginals (f1/f2) and the ratio of row marginal (g1/g2) are close to 1, while symmetry refers to whether the difference in column marginal (f1-f2) has the same sign as the difference in row marginal (g1-g2). The first paradox noted by Feinstein and Cicchetti [7] was that one gets lower kappa values despite high observed agreement [P_O_ = (x_11_+ x_22_)/N)] when the marginals are imbalanced. The second paradox is that one has higher kappa values for asymmetrical than for symmetrical imbalanced marginal totals and for imperfect versus perfect symmetry in the imbalance.

**Table 1:** Generic 2x2 table format for assessing agreement between two raters classifying N units into the same 2 categories

		**Rater B**		
		**Yes**	**No**	**Total**
**Rater A**	**Yes**	x_11_	x_12_	g1
	**No**	x_21_	x_22_	g2
	**Total**	f1	f2	N

Cicchetti and Feinstein [8] suggested resolving the paradoxes by using two separate indexes (p_pos_ and p_neg_) to quantify agreement in the positive and negative decisions; these are analogous to sensitivity and specificity from a diagnostic testing perspective.

Also trying to address the two paradoxes, Byrt *et al.*[6] discussed the effect of bias and prevalence on kappa and proposed a prevalence and bias adjusted kappa, PABAK. They also suggested that when reporting kappa, one should also report bias and prevalence indices. The bias index (BI) is defined by

$$\mathrm{BI}=\left(x_{12}-x_{21}\right)/N\text{,}$$

while the prevalence index (PI) is defined as

$$\mathrm{PI}=\left(x_{11}-x_{22}\right)/N\text{.}$$

Note that BI = 0 if and only if the marginal distributions are equal. PI ranges from -1 to +1 and is equal to zero when both categories are equally probable. Similarly, Lantz and Nebenzahl [12] proposed that one should report supporting indicators along with kappa - P_O_, a symmetry indicator, and p_pos_. Unfortunately, reporting of multiple indices is often not done.

This manuscript considers the various alternative single indexes for observer agreement in 2x2 tables, and examines their behavior under different scenarios of marginal distributions, balanced or not, symmetrical or not. It is an attempt to shed more light on how these measures address the paradoxes identified by Feinstein and Cicchetti [7], but also to examine their behavior in broader situations encountered in 2x2 tables.

## Methods

### Different agreement indices

In addition to Cohen’s kappa, we consider the following statistics: Bangdiwala’s B-statistic [13,14], Prevalence Adjusted Bias Adjusted Kappa (PABAK) [6], Aickins’s alpha [15], Andrés and Marzo’s Delta [16,17] and Gwet’s AC1-index [18].

**Figure 1:**
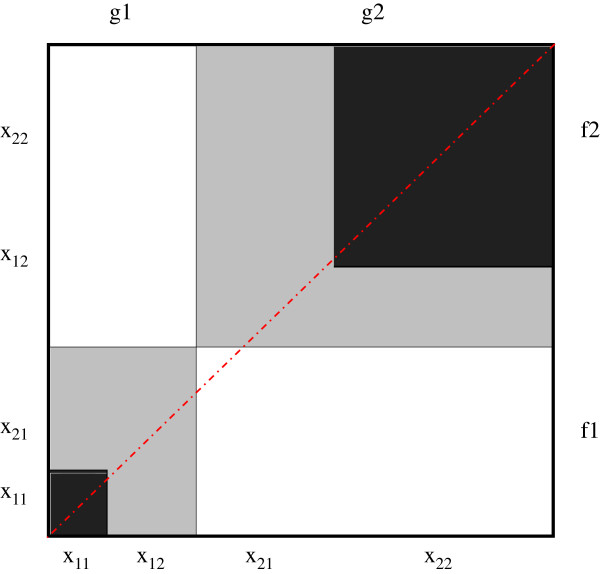
**Agreement chart for hypothetical data from Table**1**assessing agreement between two raters classifying N units into the same two categories.**

Bangdiwala [13,19] proposed the agreement chart and the corresponding B-statistic to quantify the agreement between two observers after correcting for the agreement that arises from chance alone. The agreement chart is now incorporated as a standard chart in SAS PROC FREQ, and in the VCD package in R [20] and is discussed by Friendly [21]. Details for the construction and interpretation of the agreement chart are presented by Bangdiwala and Shankar [14]. The B-statistic is defined from the agreement chart as the ratio of the sum of areas of squares of perfect agreement to the sum of areas of rectangles of marginal totals (see Figure 1), or from the 2 × 2 table as the ratio of the sums of squares of the diagonal frequencies over the sum of cross-products of the marginal totals:

$$\hat{B}=\frac{\sum_{i=1}^{q}x_{\mathrm{ii}}^{2}}{\sum_{i=1}^{q}g_{i.}f_{.i}}\text{,}$$

where x_ij_ is the cell entry of the i^th^ row and j^th^ column, *g*_*i.*_ is the i^th^ row total and *f*_.i_ is the i^th^ column total and i = 1, …, q categories [q = 2 in this paper]. The agreement chart reflects the marginal totals by rectangles and the diagonal agreement by darkened squares within the rectangles. Note that the B-statistic is a proportion of areas and thus ranges in values between 0 and 1.

The prevalence-adjusted and bias-adjusted kappa (PABAK) [6] is simply 2P_O_-1. Gwet [18] proposed an alternative Agreement Coefficient (AC1) to overcome kappa’s limitations. Gwet’s AC1-index is similar to kappa except that an adjustment is made in the expected proportion *P*_*e*_ by using the average of the marginal probabilities for each category: where

AC1^=Po-PeG1-PeGwherePeG=2×f1N+g1N2×1-f1N+g1N2=f1N+g1N2×1-f1N+g1N2+f2N+g2N2×1-f2N+g2N2

Aickin’s alpha [15] and Andrés and Marzo’s Delta [17] are statistics that consider some units are subject to classification by chance more so than others. Aickin [15] proposed a model-based estimate using maximum likelihood estimation for estimating alpha, while given the categorical latent variable, Aickin’s model can be shown to be a log-linear model within a mixture-model framework [22]. Under this approach with k = 2, Guggenmoos-Holzmann [22,23] provided a simplified formula to estimate alpha, which is given by:

$$\hat{\alpha }=\left[1-\frac{1}{\sqrt{x_{11}x_{22}/x_{12}x_{21}}}\right]P_{O}$$

Andrés and Marzo [17] proposed a different kind of model based index they called ‘delta,’ based on a multiple-choice test that measures “proportion of agreements that are not due to chance.” Delta is given by

$$\begin{array}{c} \hat{\Delta }=\frac{\left(g_{1}+1.5\right){\hat{\Delta }}_{1}+\left(g_{2}+1.5\right){\hat{\Delta }}_{2}}{N+3}\\ {\hat{\Delta }}_{i}=\frac{\left(x_{\mathrm{ii}}+0.5\right)-\left(g_{i}+1.5\right){\hat{\pi }}_{i}}{\left(g_{i}+1.5\right)\left(1-{\hat{\pi }}_{i}\right)}\\ {\hat{\pi }}_{1},{\hat{\pi }}_{2}=\left\{M\pm \left(x_{21}-x_{12}\right)-\sqrt{{\left\{M+\left(x_{21}-x_{12}\right)\right\}}^{2}-4\left(x_{21}+1\right)M}\right\}/2M\text{,} \end{array}$$

where M is the iterative numerical solution to the following equation:

$$M-2\sqrt{{\left\{M+x_{21}-x_{12}\right\}}^{2}-4\left(x_{21}+1\right)M}-\sqrt{M\left(M-4\right)}=0$$

To simplify the estimation, the authors Andrés and Femia-Marzo [16,17] proposed an asymptotic estimator by adding one to all outcomes and gave the following formula

$${\hat{\Delta }}_{a+1}=\frac{x_{11}+x_{22}+2-2\sqrt{\left(x_{12}+1\right)\left(x_{21}+1\right)}}{n+4}$$

In order to examine the behavior of the above statistics, we specify similar scenarios as Byrt *et al.*[6]*,* Feinstein and Cicchetti [7], Cicchetti and Feinstein [8]; these are provided in Table 2. The corresponding agreement charts are presented to help the reader visualize the degree of agreement, and balance and symmetry of the marginal totals. Table 2 also presents the observed agreement (P_O_), bias index (BI), and prevalence index (PI).

**Table 2:** Scenarios studied in this manuscript: Cell frequencies, marginals, proportion observed, bias and prevalence index

**Scenario**	**Type of table**	**x**_**11**_	**x**_**12**_	**x**_**21**_	**x**_**22**_	**f1**	**f2**	**g1**	**g2**	**P**_**O**_	**BI**	**PI**
**Paradox 1:**												
1	Symmetrical balance	40	9	6	45	49	51	46	54	.85	.03	-.05
2	Symmetrical imbalance	80	10	5	5	90	10	85	15	.85	.05	.75
3	Perfect symmetrical imbalance	90	5	5	0	95	5	95	5	.90	0	.90
**Paradox 2:**	*P*_*O*_*set at 0.60*											
4	Symmetrical imbalance	45	15	25	15	60	40	70	30	.60	-.10	.30
5	Asymmetrical imbalance	25	35	5	35	60	40	30	70	.60	.30	.10
6	Perfect symmetrical imbalance	40	20	20	20	60	40	60	40	.60	0	.20
7	Asymmetrical imbalance	40	35	5	20	75	25	45	55	.60	.30	.20
8	Asymmetrical imbalance	30	30	10	30	40	60	60	40	.60	.20	0
	*P*_*O*_*set at 0.90*											
9	Perfect symmetrical imbalance	85	5	5	5	90	10	90	10	.90	0	.80
10	Symmetrical imbalance	70	10	0	20	80	20	70	30	.90	.10	.50
	*P*_*O*_*low (≤50%)*											
11	Perfect symmetrical balance	25	25	25	25	50	50	50	50	.50	0	0
12	Asymmetrical imbalance	30	30	20	20	60	40	50	50	.50	.10	.10
13	Perfect symmetrical balance	20	30	30	20	50	50	50	50	.40	0	0
14	Perfect symmetrical balance	5	45	45	5	50	50	50	50	.10	0	0

## Results

### Paradox 1

**Figure 2:**
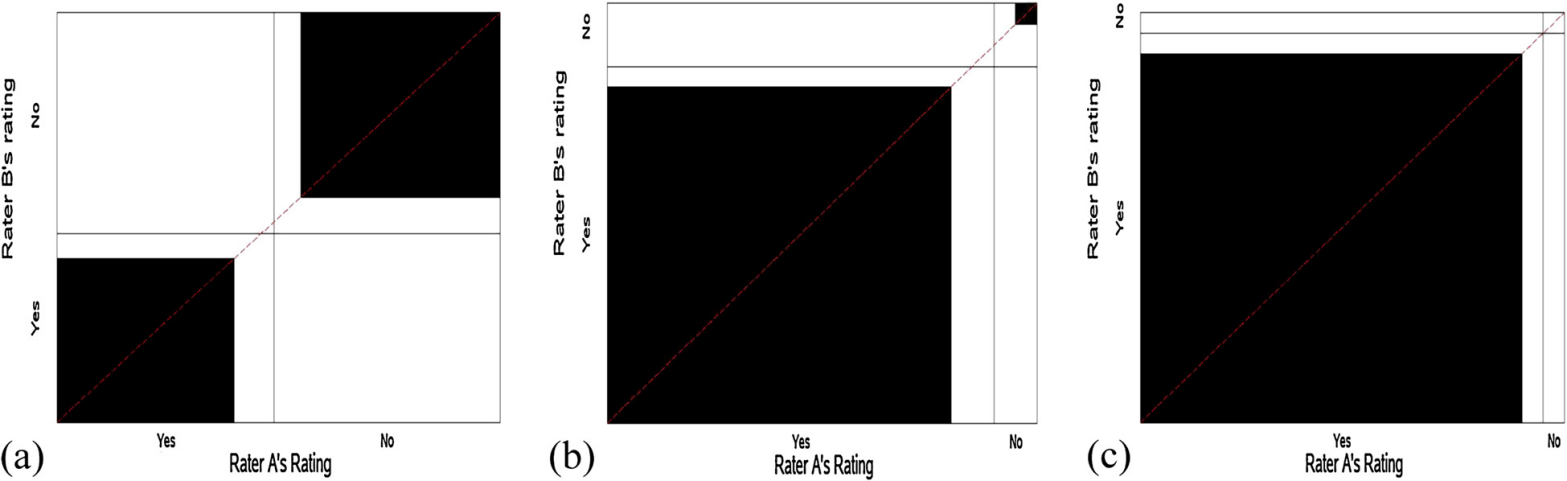
**Agreement charts (a-c) for scenarios 1-3 of Table**2**, addressing Paradox 1.**

Scenarios 1-3 address the issue of paradox 1, having a high-observed agreement but a low value for kappa. Scenario 1 has symmetrically balanced marginal totals, while scenarios 2 and 3 are symmetrical imbalances. Figure 2 presents the corresponding agreement charts for the 3 scenarios, and provides a visual image of the lack of balance. The agreement chart for scenario 1 has darkened squares of relatively the same size than the agreement charts for scenarios 2-3, within rectangles that are also close to square. The amount of darkening suggests there is a high level of agreement in all three scenarios.

We note that under symmetry, all the statistics (see Table 3) are comparable and relatively high [scenario 1]. For symmetrically imbalanced cases, we notice that when the prevalence index (PI) is large, kappa has a low value [scenarios 2 and 3]. Kappa goes as far as having a negative value for scenario 3, indicating agreement less than that due to chance. Alpha is not calculable if any cell is empty or odds ratio <1 as is the case in scenario 3. When the observed agreement is present in only one of the diagonal cells (scenario 3), AC1-index and B-statistic have values very close to the observed agreement P_O_.

**Table 3:** Estimates of proportion observed, proportion expected and agreement measures, by scenarios

**Scenario**	**Type of table**	**P**_**o**_	**P**_**e**_	$\hat{k}$	$\hat{B}$	***PABAK***	$\hat{\mathrm{AC}1}$	$\hat{\alpha }$	$\hat{\Delta }$	${\hat{\Delta }}_{a+1}$
**Paradox 1:**										
1	Symmetrical balance	.85	.50	.70	.72	.70	.70	.70	.68	.68
2	Symmetrical imbalance	.85	.78	.32	.82	.70	.81	.55	.69	.68
3	Perfect symmetrical imbalance	.90	.905	-.05	.895	.80	.89	-	.78	.77
**Paradox 2:**	*P*_*O*_*set at 0.60*									
4	Symmetrical imbalance	.60	.54	.13	.41	.20	.27	.15	.21	.20
5	Asymmetrical imbalance	.60	.46	.26	.4	.20	.21	.33	.32	.31
6	Perfect symmetrical imbalance	.60	.52	.17	.38	.20	.23	.18	.19	.19
7	Asymmetrical imbalance	.60	.475	.24	.42	.20	.23	.32	.32	.31
8	Asymmetrical imbalance	.60	.48	.23	.38	.20	.20	.25	.24	.24
	*P*_*O*_*set at 0.90*									
9	Perfect symmetrical imbalance	.90	.82	.44	.88	.80	.88	.68	.78	.77
10	Symmetrical imbalance	.90	.62	.74	.85	.80	.84	-	.83	.82
	*P*_*O*_*low (≤50%)*									
11	Perfect symmetrical balance	.50	.50	0	.25	0	0	0	0	0
12	Asymmetrical imbalance	.50	.50	0	.26	0	-.11	0	.01	.01
13	Perfect symmetrical balance	.40	.50	-.20	.16	-.20	-.20	-	-.19	-.19
14	Perfect symmetrical balance	.10	.50	-.80	.01	-.80	-.80	-.18	-.77	-.77

**Figure 3:**
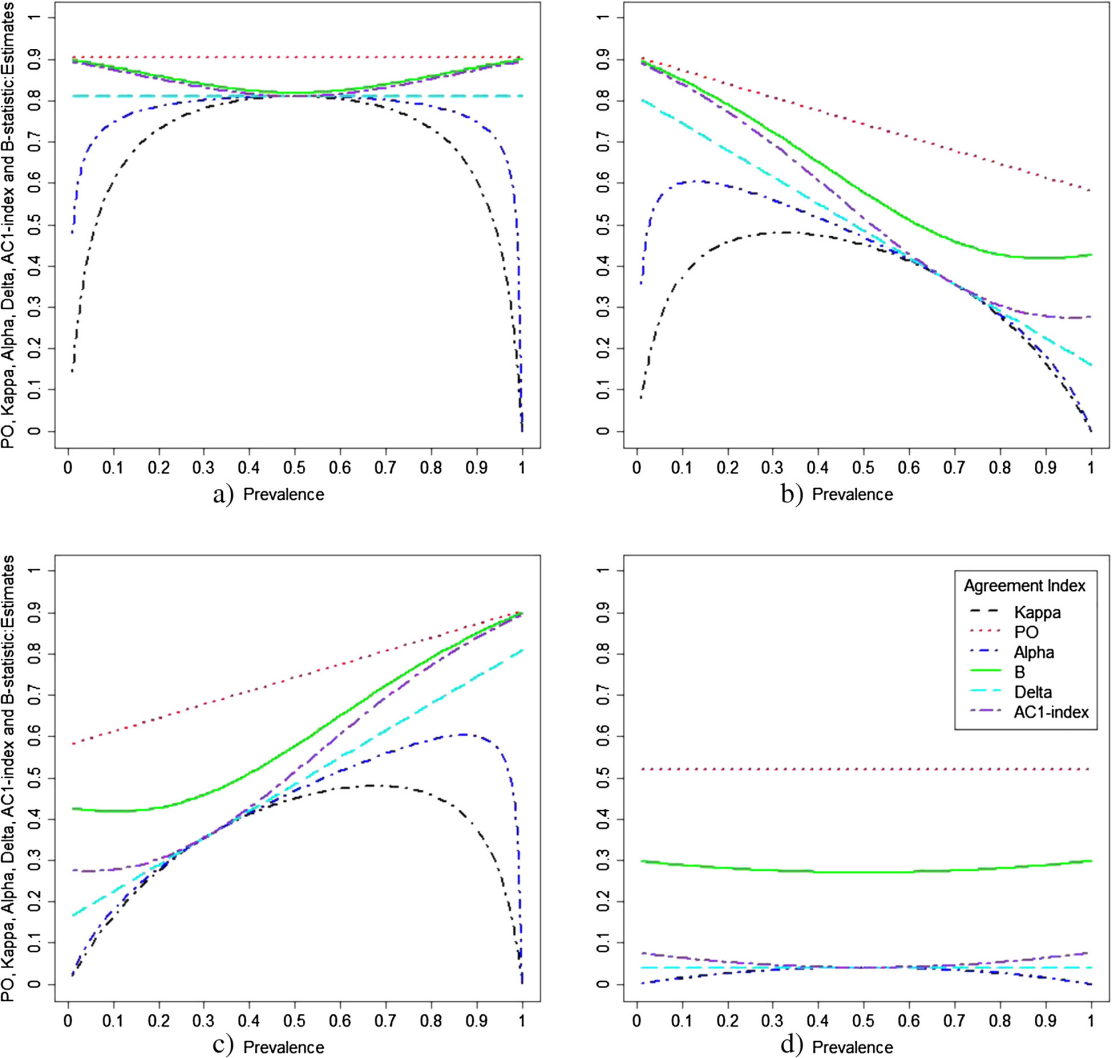
Measures of agreement as a function of prevalence, for (a) both sensitivity and specificity set at 95%, (b) sensitivity of 70% and specificity of 95%, (c) sensitivity of 95% and specificity of 70%, and (d) both sensitivity and specificity set at 60%.

In order to better understand the role of prevalence in Paradox 1, we also examined the influence of prevalence, sensitivity and specificity on the various statistics (Figure 3a-d). We considered four scenarios with varying prevalence (a) both sensitivity and specificity set at 95% (b) sensitivity of 70% and specificity of 95% (c) sensitivity of 95% and specificity of 70% and (d) both sensitivity and specificity set at 60%. Under scenario (a) with high sensitivity and specificity, all the statistics are influenced by prevalence, B-statistic and AC1-index behave similarly and are less affected by prevalence indices closer to 0 and 1. Thus, they adequately address paradox 1. When the sensitivity and specificity are different, the B and AC1-index behave better than the others. When the sensitivity is smaller compared to specificity (Figure 3b), the estimates of B and AC1-index are closer to the observed agreement P_O_ with small prevalence while when the sensitivity is larger than the specificity (Figure 3b), the B and AC1-index are closer to the observed agreement at higher prevalence. When both sensitivity and specificity are closer to 50% (Figure 3d) only the B-statistics behaves well. All the statistics except for delta statistic behave in a quadratic fashion as the prevalence changes under all scenarios. Delta behaves in a strict linear form.

### Paradox 2

**Figure 4:**
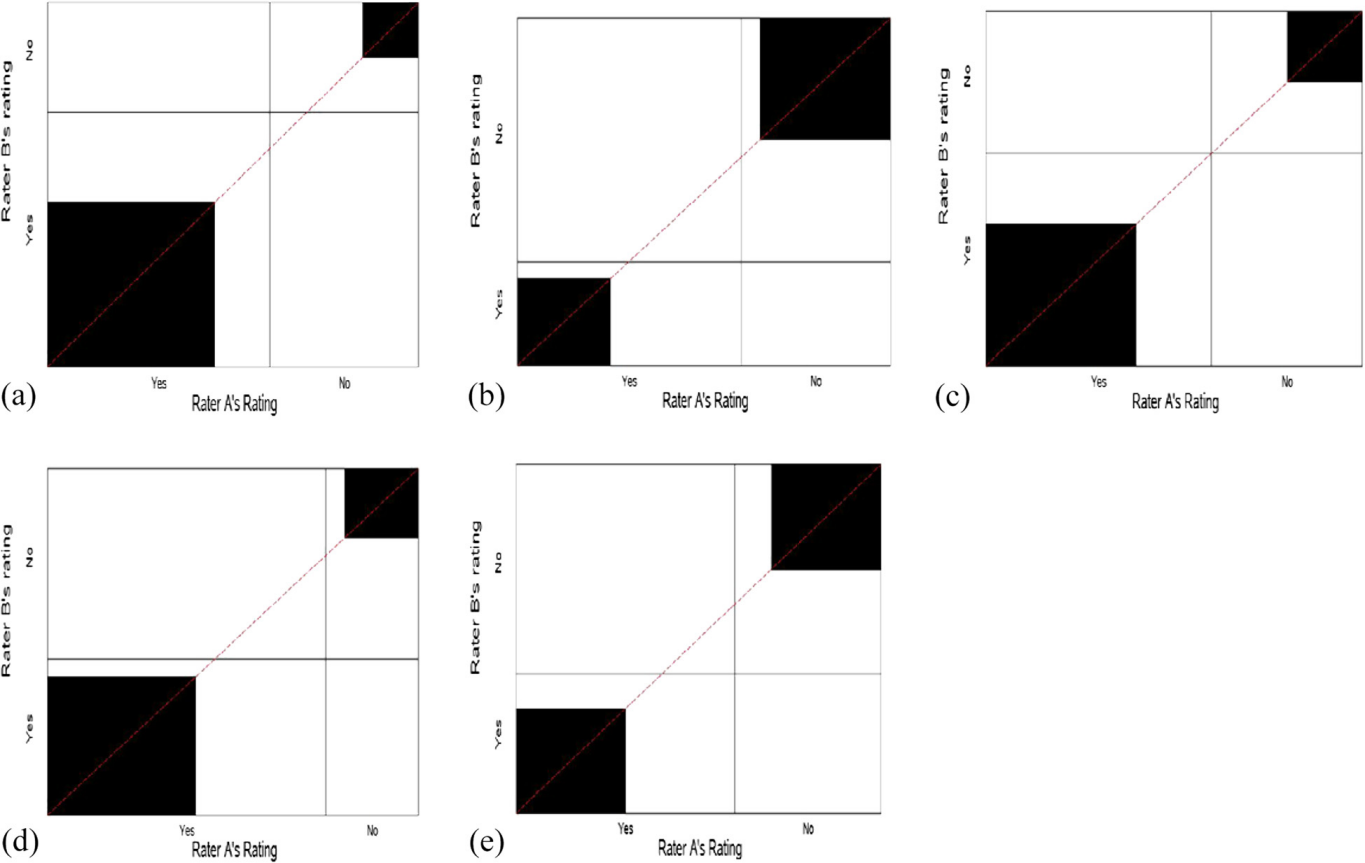
**Agreement charts (a-e) for scenarios 4-8 of Table**2**, addressing Paradox 2.**

**Figure 5:**
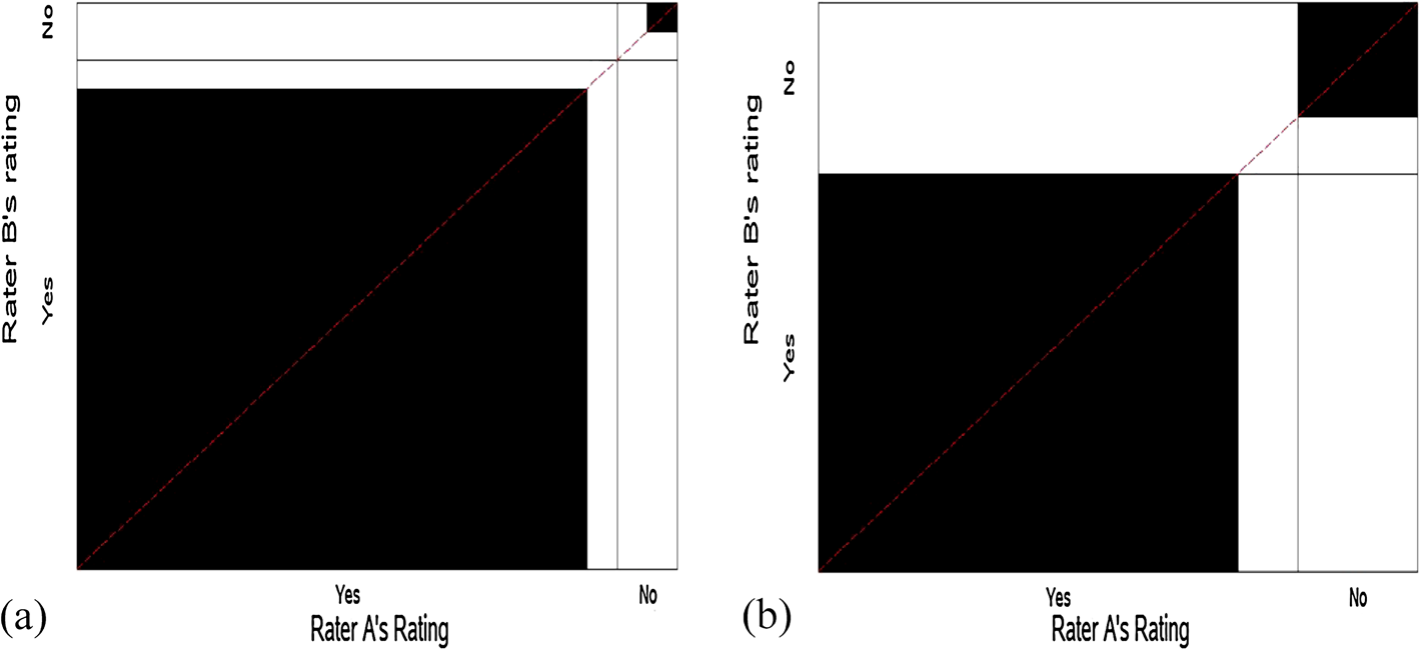
**Agreement charts (a-b) for scenarios 9-10 of Table**2**, addressing Paradox 2 with high P**_**O**_**.**

In order to address the second paradox, we consider scenarios with symmetrical versus asymmetrical imbalanced marginal totals (scenarios 4-8 with same P_O_ = 0.60) and scenarios with perfect symmetrical imbalance versus imperfect symmetrical imbalance (scenarios 9-10 with same P_O_ = 0.90). Figure 4 presents the corresponding agreement charts for scenarios 4-8, in order to aid the reader in visualizing differences in amount of symmetry when imbalanced, but the observed agreement P_O_ is constant. We note that asymmetry results in the diagonal line not coinciding with the vertex of the rectangles, and the direction of the asymmetry depends on the direction of the bias: negative bias index has a diagonal below the vertex and positive bias index has a diagonal above the vertex. Perfect symmetry is when there is no bias and thus the vertex meets the diagonal line. Figure 5 shows the corresponding agreement charts for scenarios 9-10 in order to provide a visual of imperfect versus perfect symmetrical agreement for a high value of P_O_. We notice a larger area of darkened squares, and that imperfect symmetry under high agreement forces one of the off-diagonal cells to be zero.

Kappa, alpha and delta have higher values of agreement for asymmetrical imbalance (scenarios 5 and 7) than for symmetrically imbalanced marginal totals (scenarios 4 and 6), contrary to what is desired. The B-statistic behaves slightly better, with lower values for asymmetry (comparing scenario 4 to 5), and despite having higher values for symmetry than for asymmetry in scenarios 6 versus 7, it is not as discrepant as the other statistics. This trend was similar in the AC1-index. Comparing the degrees of symmetry (scenarios 9-10), we expect that perfect symmetrical imbalances (scenario 9) should have higher agreement than imperfect symmetrical imbalances (scenario 10). PABAK does not change with changes in prevalence or bias since it is a simple function of P_O_ (scenarios 4-10). We note that kappa and delta have higher values of agreement for imperfect versus perfect symmetry, while the B-statistic and AC1-index behave as one would prefer (scenario 9 vs. 10). B-statistic and AC1-index perform better than the other statistics when P_O_ is larger (scenarios 9-10 vs. scenarios 4-6). When the bias index is greater or equal to the prevalence index (scenarios 1, 5, 7, 8, 11, 12, 13 & 14), the AC1-index is almost same as the PABAK. The slightly poor performance of B-statistic for lower P_O_ values is seen when the bias index is greater than the prevalence index (scenarios 4 vs. 5 and 6 vs. 8). In scenarios 4-8 with P_O_ = 0.60, most indices perform poor, with values substantially lower than P_O_; however, the B-statistics is closer to P_O._ Thus, B-statistic resolves paradox 2 when P_O_ is large and comes closer than the other statistics when P_O_ is smaller.

**Figure 6:**
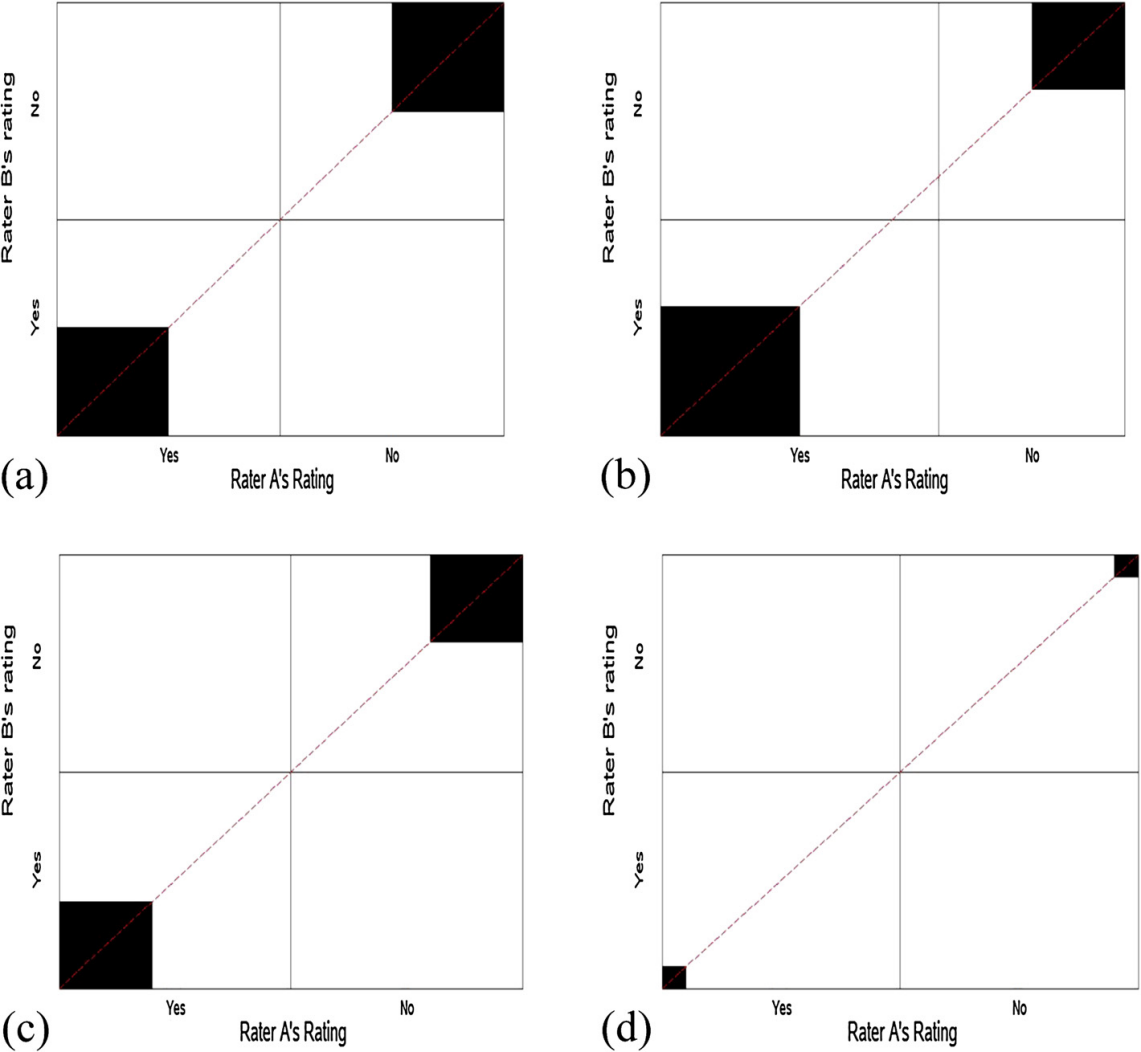
**Agreement charts (a-d) for scenarios 11-14 of Table**2**, addressing P**_**O**_ **< =0.5.**

Scenarios 11-14 examine the behavior of the statistics when P_O_ ≤ 0.50 (Figure 6a-d). This situation can arise in social or behavioral studies, where there is increased difficulty in classifying the units/individuals. We note that under these scenarios, all statistics except B-statistic show no agreement beyond chance. The B-statistic behaves as the square of P_O_ and leads to a better interpretation.

## Discussion

\While all statistics examined are affected by lack of symmetry and by imbalances in the marginal totals, the B-statistic comes closest to resolving the paradoxes identified by Fienstein and Cicchetti [7] and Byrt *et al.*[6]. Alpha behaves similarly to kappa and is thus greatly affected by the imbalances and lack of symmetry in the marginal totals. The B-statistic and AC1-index were less affected by the imbalances and lack of symmetry in the marginal totals, and were also less sensitive to extreme values of the prevalence. Delta behaves somewhat intermediate between B-statistic and kappa. Delta uses an arbitrary category for calculation in the 2x2 scenario, which makes it not realistic; but the asymptotic estimation with increment of one is closer to non-asymptotic estimates. The B-statistic came closer to resolving both paradoxes than any of the other indices, and thus we recommend use of the B-statistic when assessing agreement in 2x2 tables. However, we note that as Nelson and Pepe [10] suggest, visual representations ‘provide more meaningful descriptions than numeric summaries’ (p. 493), and thus we recommend additionally providing the corresponding agreement chart to illustrate the agreement as well as constraints from the symmetry and balance of the marginal totals and cell frequencies. The B- statistic is easy to calculate and along with the agreement chart, it provides interpretations of the agreement pattern as well as the disagreement pattern between the raters.

## Conclusions

The B-statistic behaved better under all scenarios of marginal distributions studied, balanced or not, symmetrical or not, as well as with varying prevalences, sensitivities and specificities than the other measures. We recommend using B-statistic along with its corresponding agreement chart as an alternative to kappa when assessing agreement in 2x2 tables.

## Competing interests

The authors declare that they have no competing interests.

## Authors’ contributions

VS was involved in conceptualization, literature search, writing, data analysis and creating charts for the study. SIB was involved in conceptualization, writing and data interpretation of the study. Both authors read and approved the final manuscript.

## Pre-publication history

The pre-publication history for this paper can be accessed here:

http://www.biomedcentral.com/1471-2288/14/100/prepub
